# Circular Economy of Construction and Demolition Waste for Nanocomposite Cement: XRD, NMR, Vickers, Voltammetric and EIS Characterization

**DOI:** 10.3390/nano14151239

**Published:** 2024-07-23

**Authors:** Roxana Rada, Daniela Lucia Manea, Simona Rada, Radu Fechete

**Affiliations:** 1 Faculty of Civil Engineering, Technical University of Cluj-Napoca, 400020 Cluj-Napoca, Romania; roxana.rada@ccm.utcluj.ro (R.R.); daniela.manea@ccm.utcluj.ro (D.L.M.); 2 Physics and Chemistry Department, Technical University of Cluj-Napoca, 400020 Cluj-Napoca, Romania; radu.fechete@phys.utcluj.ro; 3 National Institute for Research and Development of Isotopic and Molecular Technologies, 400293 Cluj-Napoca, Romania

**Keywords:** construction and demolition waste, recycling, nanocomposites, cement, XRD, NMR, EIS, CV, SLV, Vickers

## Abstract

In this paper, we present the structural, mechanical and electrical properties of composite cement materials that can be widely used as substituent for cement. We start with the characterization of a composite cement sample using an analysis of X-ray diffraction (XRD) and nuclear magnetic resonance (NMR) spectra. The measurements of the Vickers hardness, cyclic and sweep linear voltammetry and electrochemical impedance spectroscopy (EIS) of composite cement materials were also recorded. This study compared the effect of the different nanocomposites added to cement on the mitigation of the alkali–silica reaction, which is responsible for the swelling, cracking and deleterious behavior of the material. The enhancement in Vickers hardness was more pronounced for composite cement materials. In contrast, the values of Vickers hardness decreased for the composite cement containing mortar and the control sample, suggesting that the long-term performance of cement was compromised. In order to obtain information about the bulk resistance of the composite cement material, electrochemical impedance spectroscopy (EIS) data were employed. The results suggest that for composite cement materials, there is an improvement in bulk electrical resistance, which can be attributed to the lower amounts of cracks and swelling due to lower expansion. In the control sample, a reduction in the bulk resistance suggests the formation of microcracks, which cause the aging and degradation of the material. The intersection of arcs in the EIS spectrum of the mixed composite cement sample gradually increased by an alkaline exposure of up to 21 days and finally shifted towards a low value of high frequency with an increase in alkaline exposure of up to 28 days.

## 1. Introduction

The fabrication of cement is responsible for carbon dioxide emissions above 8% in the environment and about 3100 MJ/ton of energy consumption in clinker production [1,2].

The application of supplementary materials such as additives in cementitious materials has been receiving significant attention nowadays for reducing carbon emissions. Some examples of supplementary cementitious materials used in the form of nanoparticles are nano-TiO_2_, nano-CaCO_3_, carbon nanotubes, nano-silica, and nano-aluminate [3,4,5]. These additives can enhance the performance of cement-based materials and promote the formation of hydration products.

Compared with the abovementioned information, synthetic calcium–silicate–hydrate (C-S-H) materials have demonstrated a pronounced effect in improving the age performance of cement materials [6]. A 5–10% level of silica fume, used as a replacement in cement paste, produces high-performance concrete and improves their strength and durability. Mixes with silica fume can react with Ca(OH)_2_ to yield an increase in C-S-H chain length and modify the microstructure of cement paste [7].

In accordance with global directives towards resource conservation, the circular economy and the development of sustainable concrete structures, recycled fibers were tested for their capability to maintain their structural performance. Expensive and nonbiodegradable material resources such as steel, glass, carbon polypropylene, polyethylene and polyvinyl chloride provide concrete with an improved strengthening performance [8]. The implementation of these natural materials in lightweight cement mortar is feasible because of the preserved mechanical properties and because they have a higher efficiency in acoustic insulation [9]. Their reuse provides several advantages, including an improvement in the mechanical response of concrete, a decrease in environmental waste and socioeconomic impact.

Despite the aforementioned advantages, their usefulness in cementitious materials is limited by their low durability and their degradation in alkaline environments of the cement matrix [10].

The incorporation of nanomaterials such as nano-silica and graphene oxide in concrete has drawn great attention because they can significantly enhance early-age properties and do not compromise the long-term performance of concrete [11].

Fly ash concrete has been used in building construction due to its enhanced durability and cost-saving and environmental protection qualities [12]. The main disadvantage of the replacement of cement with fly ash is the low early-age strength of concrete due to a slow pozzolanic reaction [13]. The pozzolanic reaction is defined as the chemical reaction between reactive silica or alumina in fly ash particles and Portlandite (calcium hydroxide) formed during the cement hydration process in the presence of water at ambient temperature.

The use of calcined clays as a replacement for cement in concrete is a promising approach because of their high pozzolanic reactivity due to the chemical reaction of amorphous aluminosilicate phases with calcium hydroxide to form additional hydrates such as calcium silicate hydrate [14].

Some studies reported that pozzolanic materials such as waste glass powder and nano-SiO_2_ can mitigate the alkali–silica reaction and decrease its expansion [15]. The alkali–silica reaction is responsible for the deleterious behavior of concrete due to the reactions between the alkaline pore solution and the amorphous or metastable forms of silica in aggregates. As a result, cracks in the vicinity of the reactive species can be initiated and developed to deteriorate the cement material.

The exploitation of construction and demolition waste in industrialized countries is considered very attractive regarding the environmental and economic benefits from their reutilization.

The circular economy comprises a novel reformative framework that aids in optimizing the consumption of raw materials and ensures the value of materials throughout their lifecycle. It prevents the generation of excess waste, hence preserving natural resources while demonstrating that everything that is made can be recycled, reprocessed or reused.

Construction and demolition waste represents about half of the total amount of municipal solid waste generated in European countries, occupying a large storage space and causing pollution, which are becoming serious problems. This includes all the waste produced during the construction, renovation and demolition activities of buildings, roads and other structures.

The deepening of the recycling and reuse routes of construction and demolition waste is an attractive challenge accompanied by a reduction in pollution and storage problems and would allow for an economy of natural resources to become an advantageous solution from year to year.

Municipal waste contains the following types of construction and demolition waste: (i) concrete, bricks, ceramic waste; (ii) wood waste, glass, plastic; (iii) asphalt and tar waste; (iv) metal scraps; (v) remains of excavations such as soil, stones, gravel; (vi) insulating material waste; (vii) mixtures of construction and demolition waste.

C&D waste comprises the largest waste stream and high recovery rates, suggesting that the building sectors are highly circular. The inspection of management practices reveals the low recovery of the C&D waste that relies on the use of recycled aggregates of road pavements and sub-base. These actions degrade significantly the technical and economic value and do not solve the C&D waste problem. Actions inspired by the circular economy can help the achievement of objectives of waste policy, such as the increase in quantities and qualities of recycled C&D waste.

The concept of a circular economy is associated with the maintenance of the values of materials and products over time and reducing the environmental impact of the raw materials, energy and environmental impacts (resource extraction, emissions and waste management). Circular waste management implies the redesign of the material cycle process such that there is economic prosperity and social benefits.

Costs are key for the implementation of waste management systems. The benefit–cost analysis is important to estimate the economic feasibility of construction waste minimization. For the reuse and recycling of C&D waste, a net benefit of 2.5% from the total budget was reported [16].

In this paper, the effect of adding varied nanocomposites based on construction and demolition waste in the cement material were investigated by XRD, NMR and Vickers hardness data. The ^1^H NMR relaxation data were used to evaluate the water reservoirs in pore structure. The impact of alkali–silica reactions on composite–cement materials was evaluated by bulk electric resistances. The use of C&D waste for the production of new construction products brings benefits for the conservation of resources and the reduction in the amount of waste. The replacement of Portland cement with composites containing C&D waste is expected to produce a sustainable construction material.

## 2. Experimental Procedure

### 2.1. Preparation of Composites

Used raw materials in this synthesis are construction and demolition (C&D) waste, such as broken glasses, lime, mortar, plaster, autoclaved aerated concrete and brick.

In a capsule with sodium hydroxide solution of a concentration of 1M, broken glassy powders were introduced and agitated mechanically at 40 °C. After the partial dissolving of the glassy powders, a solution of chloride acid at a concentration of 1 M was added, in addition to lime powder. The temperature synthesis was modified to 100 °C for 10 min and then was kept at 350 °C for 30–60 min.

The described wet synthesis method was also applied for the preparation of other types of composites based on varied C&D waste, namely brick, autoclaved aerated concrete, mortar or plaster powder. A composite containing a mix of these five wastes was synthesized.

### 2.2. Preparation of Composite–Cement

The composite–cement samples were prepared using gray Portland cement, composites and water. The amounts of composite and water in the cement mass were 2.5 weight % and 30%, respectively. The composite–cement mixtures were cast in molds with dimensions of approximatively 30 × 20 × 10 mm and kept in air.

In Table 1 is summarized the compositions of the composites and composite–cement samples.

### 2.3. Methods

X-ray diffractograms were recorded on composite–cement powders using the Regaku diffractometer with a radiation of 1.54 Å. The Match! Software with Version 1.0 was used for the identification of the crystalline phases.

FTIR spectra were recorded using the JASCO 6200 FTIR spectrometer (Tokyo, Japan) using a resolution of 4 cm^−1^. The powder sample was mixed with KBr at the mass ratio of 1:150 and then pressed with a load of 5 tons/cm^2^ to produce transparent disks.

The measurements of NMR spectroscopy were realized using a low field Bruker Minispec NMR spectrometer (Bruker, Karlsruhe, Germany) operating at 19.69 MHz proton frequency.

The Vickers micro-hardness was determined using a Nova micro-durimeter (InnovaTest, Maastricht, The Netherlands) by indentation method having a penetrator which was operated with a load of 0.3 kgF at an interval of 15 min.

Electrochemical impedance spectroscopy (EIS) was used to characterize the change in bulk electrical resistance of composite–cement materials during the accelerated alkali–silicate reaction test at 3, 7, 14 and 28 days. EIS, cyclic voltammetry (CV) and sweep linear voltammetry (SLV) measurements were conducted by AutoLab PGSTAT 302 N (EcoChemie, Utrecht, The Netherlands) equipment and software Nova version 1.11. All measurements were performed in an electrochimic cell with three electrodes using a calomel electrode as the reference electrode, composite–cement material as the working electrode and platinum electrode as the contraelectrode.

## 3. Results and Discussion

### 3.1. X-ray Diffractograms

**Figure 1:**
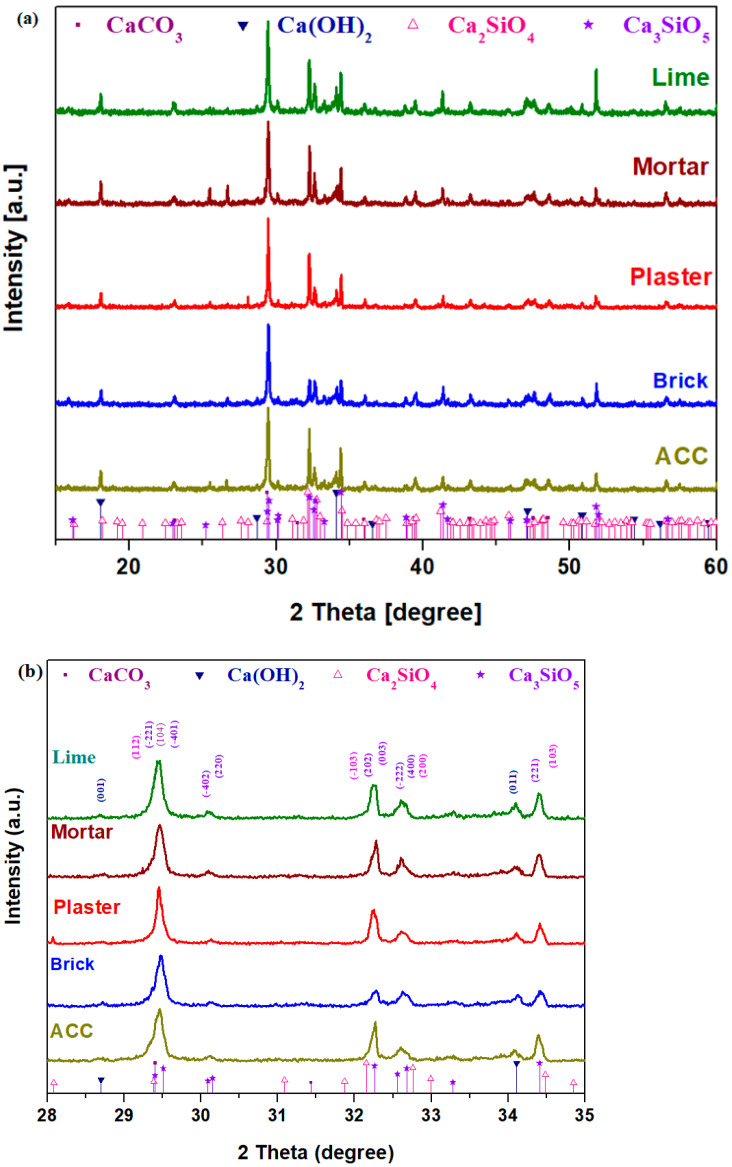
X-ray patterns of the composite—cement materials in the region between (**a**) 10–60 degrees and (**b**) 28–35 degrees. The Miller indices are also inserted in the subfigure.

The structures of prepared composite–cement materials were observed by analysis of X-ray diffraction. XRD results of the composite–cement samples are depicted in Figure 1. All patterns indicate the presence of four crystalline phases, namely tricalcium silicate, Ca_3_SiO_5_ ≡ 3CaO·SiO_2_, dicalcium silicate, Ca_2_SiO_4_ ≡ 2CaO·SiO_2_, CaCO_3_ and Ca(OH)_2_ crystalline phases. The amounts of the di- and tri-calcium silicate crystalline phases increase in the lime and ACC composite–cement and they were reduced in the brick sample when compared with their counterparts.

Ca_3_SiO_5_ is known in the composition of new Portland cement and has superior physicochemical and biological properties [17]. The dicalcium silicate was developed to reduce the clinker agent and the energy consumption during manufacturing of the cement [18].

**Figure 2:**
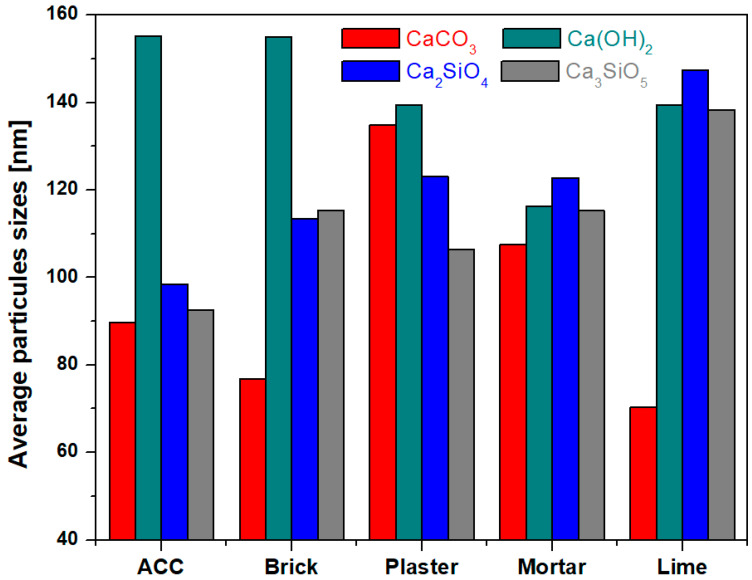
Compositional evolution of average particles sizes from composite–cement.

It can be found from Figure 2 that the different particles sizes do not change much and their values are varied between 70 and 155 nm. The characteristic particles sizes of the cement matrix containing mortar are below 125 nm, and it can be considered a finer composite.

### 3.2. NMR Spectra

**Figure 3:**
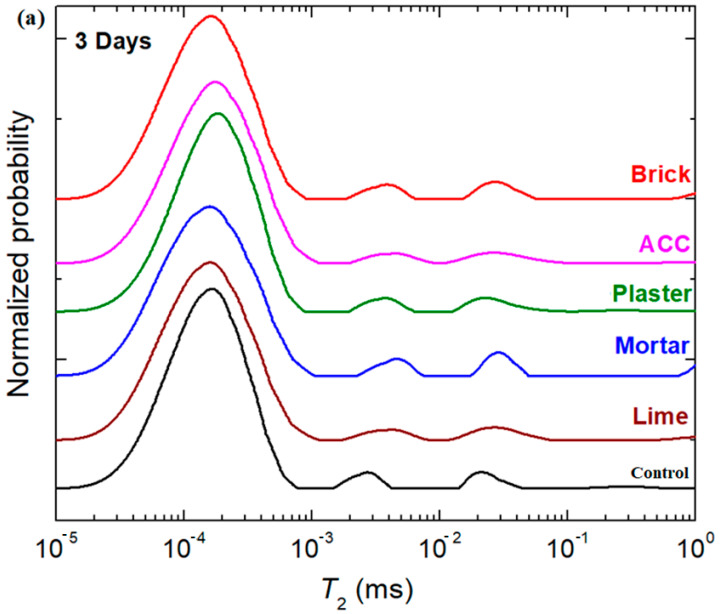

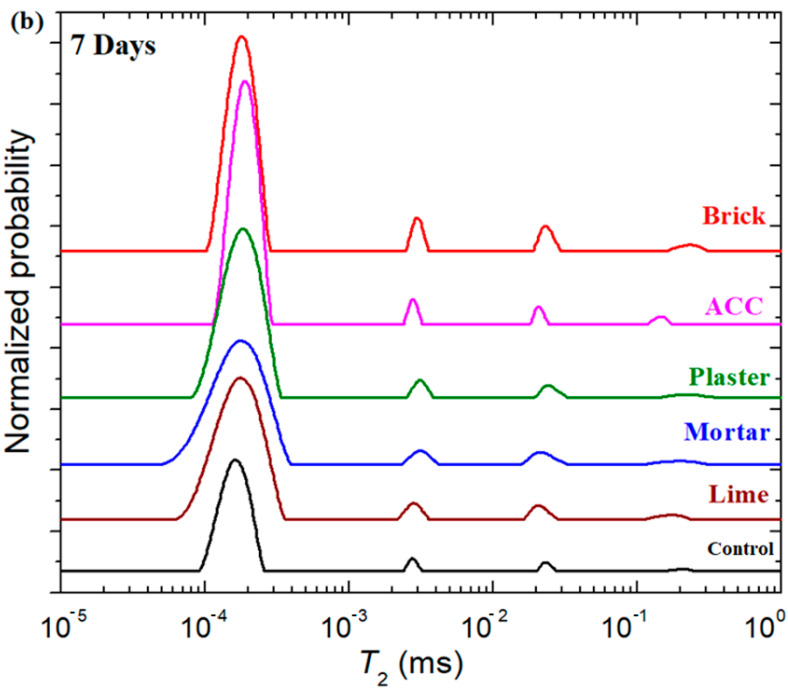

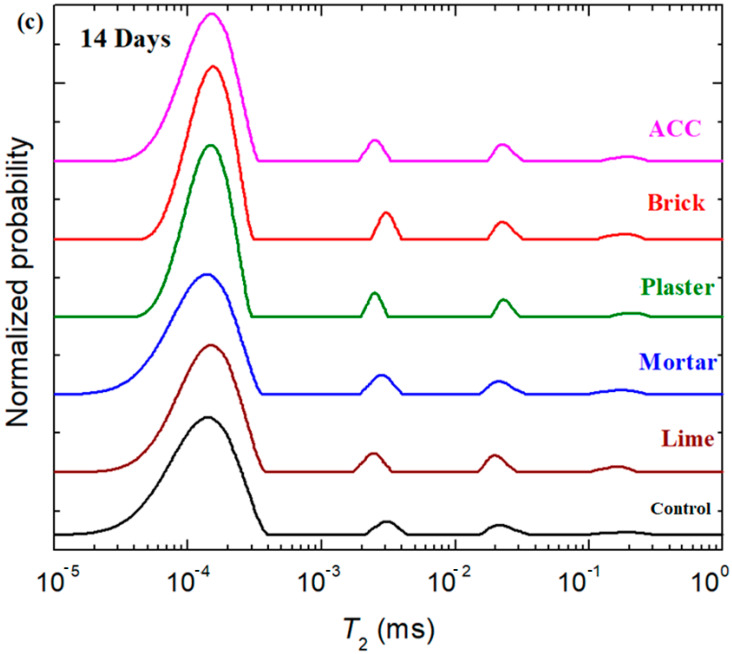

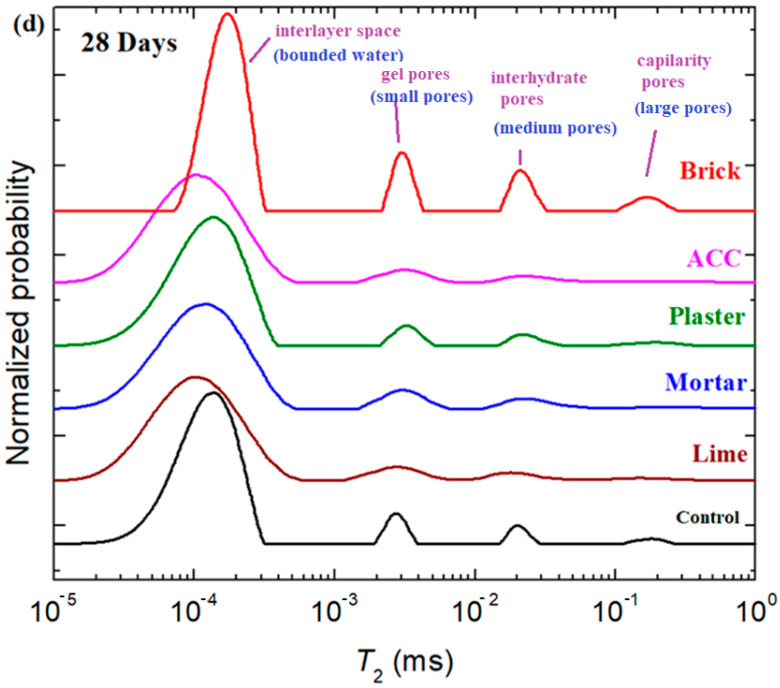
NMR spectra of the composite–cement samples recorded at (**a**) 3, (**b**) 7, (**c**) 14 and (**d**) 28 days after their preparation. The types of water reservoirs are also inserted.

The NMR spectra of the composite–cement materials during hydration performed at 3, 7, 14 and 28 days after their preparation are presented in Figure 3. NMR spectra allow us to unambiguously distinguish the water reservoirs corresponding to the evolution of the pores. Four water reservoirs are observed, which from the shorter *T_2_* relaxation time, are labelled bounded water, water in small pores, water in medium pores and water in larger pores [19,20]. The interpretation of ^1^H NMR relaxation data in cements is also based on the observation that the NMR signal can be categorized by their size and can be resolved into four discrete populations of water. The first two of these components are assigned to interlayer water within the C-S-H and to C-S-H gel pore water. The last two components are attributed to water in nanoscale inter-hydrate spaces and to large capillarity pores and micro-cracks [21]. They are defined as the free water within the paste. The evolution of the water content at these positions from *T_2_* distributions is first considered because this will directly influence the hydration reaction and pore structures.

**Figure 4:**
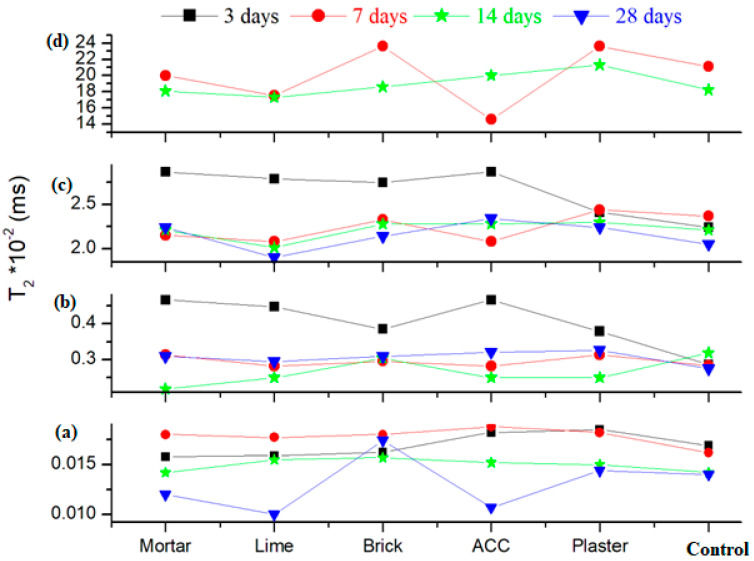
The relaxation time distributions of mobile water from NMR data of composite–cement materials at. (**a**) interlayer peak, (**b**) gel pore peak, (**c**) inter-hydrate pore peak, and (**d**) capillarity pore peak at 28 days after their preparation.

Figure 3a shows the T2 distribution map of composite–cement pastes after 3 days of hydration with initial water/cement ratios of 0.3. In Figure 4, it can be seen that the position of the interlayer peak for the ACC and plaster samples shifts to higher T2 relaxation times, indicating better mobility of the material components compared with the control cement. The position of the peaks assigned to the interlayer and gel pores moves to longer T2 times. The capillarity water signal is not evidenced in the T2 distribution map of different pastes. This suggests that the water is consumed from the capillarity pores during hydration.

At 7 and 14 days after preparation, all water reservoirs are well defined (see Figure 3b,c). The position of the bounded water signal moves slowly towards higher T2 relation times by adding composites in the cement for comparison with the control sample, indicating the mobility of the components in the material.

From day 7 to day 14, the water reservoir peaks, corresponding to positions associated with bounded water and water from small, medium and large pores, are migrated towards smaller T2 values, indicating that the composite–cement materials become rigid. Higher amounts of water reservoirs were observed in the brick and ACC samples after 7 days and in the brick sample at 28 days after preparation.

The T2 distributions measured at 28 days for brick composite–cement indicate that the water content is drastically increased in all four water reservoirs. Other composite–cement materials show an increase in the rigidity degree compared to the control sample and its analogues in varied early stages of hydration because the water content at the top position is decreased by drying. The position of the interlayer peak for the ACC and plaster samples shifts to higher T2 relaxation times, indicating better mobility of the material components compared with the control cement.

### 3.3. Vickers Hardness Data

The alkali–silica reaction (ASR) is one of the challenging problems related to the durability of the composite–cement material. To mitigate the alkali–silica reaction effect, a portion of cement was replaced with composite. The mitigation effect of composite on the alkali–silica reaction is related to the dilution of alkalinity, consumption of calcium hydroxide and alkali fixation by calcium silicate hydrates, C–S–H, which yield to the decrease in alkalinity in the pore solution.

In order to understand the effects of composites on the alkali–silica reaction in the composite–cement at advanced ages one year after their preparation, we determined the Vickers hardness.

**Figure 5:**
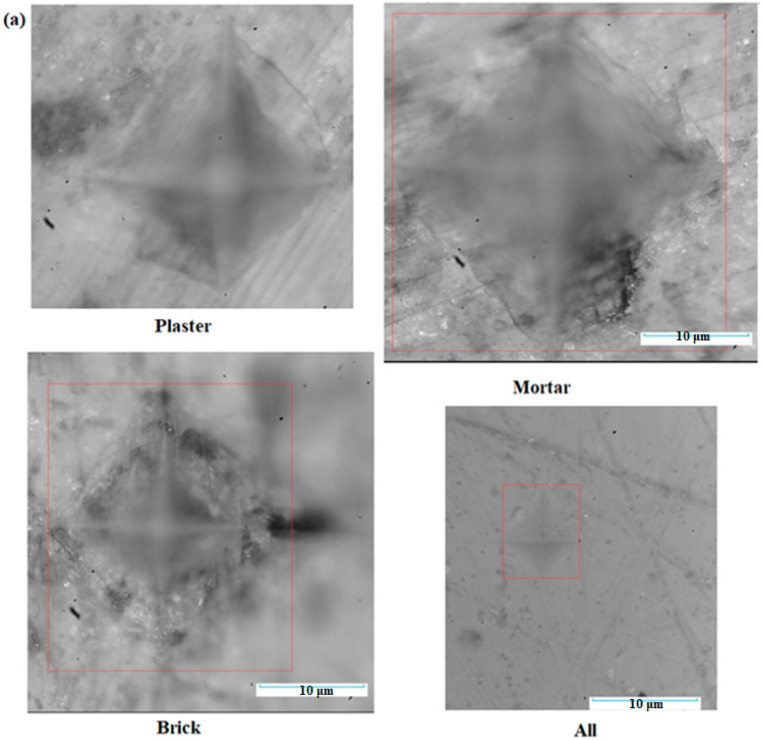

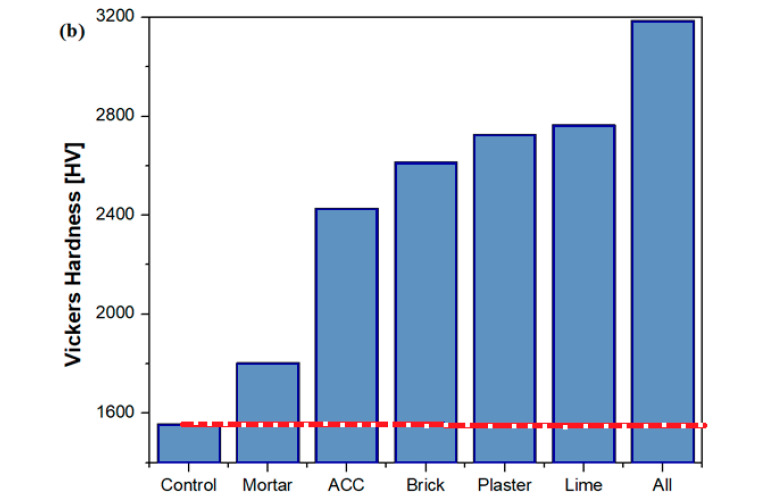
(**a**) Microscopic images after indentation (in the red square is shown the pyramidal area of the indentation) and (**b**) compositional dependence of Vickers hardness distributions of composite–cement materials (with red dashed line indicating the Vickers value of the control sample).

The microscopic micrographs after indentation and the compositional evolution of the Vickers values of composite–cement materials are depicted in Figure 5. The microscopic analysis indicates heterogeneous regions with microcracks. A good performance of the microstructure can be observed for the All sample containing five types of C&D waste.

An increase in the Vickers hardness values was found for all composite–cement materials when compared with the control sample. For the All sample, the Vickers hardness had the highest value and the micrograph after indentation is more compact. These results confirmed that mechanical properties of cement were improved by doping with composites originating from waste.

### 3.4. EIS Measurements

It is known that the cement matrix is an ionic conductor [22]. The alkali–silica reactions occur between hydroxide ions and accompanying soluble alkali ions, such as Na^+^ and K^+^ present in the interstitial solution of the concrete and aggregates. These processes generate swells and cracks, which will affect the concrete because it will initiate a reduction in the mechanical properties (compressive strength, flexural strength, change in the elastic module) and the life cycle of the concrete. When the aggregates contain a sufficient amount of silica (amorphous or poorly crystallized silica), they are vulnerable and can be mostly modifiable because they react with hydroxide ions in the hyperalkaline interstitial solution (dominated by KOH and NaOH, pH = 13.5) contained in the porosity of the hardened cement paste.

The impacts of the alkali–silica reaction on composite–cement and control cement were also evaluated with electrochemical impedance spectroscopy measurements. The electrochemical impedance method is a modern study of spectroscopy of the processes that take place in an electrochemical cell. The EIS analysis measures resistance (R), capacitance (C) and inductance (L) by monitoring the current response while a voltage (in alternative current) is applied to an electrochemical cell.

Electrically, the cellules behave as an ohmic resistor in direct current and as an impedance in alternating current. This consists of the perturbation of the system with an alternating signal superimposed on the direct current feeding the normal cell and measuring the response. Thus, the cell can be connected in an alternating current bridge that allows the impedance to be determined.

The equivalent electric circuit of a cellule can be represented by different schemes. The cell has an ohmic resistance due to the solution, to which are added those circuit elements present in the electrical double layer of the electrode interface. The total impedance of the cell, Z, is the expression of a series of combinations of resistances, R, and capacities, C. These two elements intervene in the real impedance, Z_Re_ (where Z_Re_ = R), and imaginary impedance, Z_Im_ (where Z_Im_ = 1/ωC). Regarding the processes in the cellules, the representation of the imaginary part of the complex impedance, Z_Im_, can be extracted as a function of the real part of complex impedance, as well as Z_Re_ for varied values of the frequency, ω. The bulk resistance, R_b_, of the electrolytes is inversely proportional to the conductivity, σ (R_b_ = 1/σ), of the materials.

**Figure 6:**
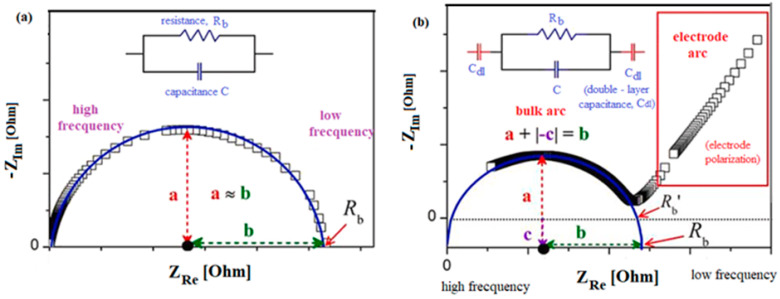
EIS spectrum for (**a**) perfect and (**b**) depressed semicircle. The equivalent circuits are also shown.

The EIS spectra can separate and quantify the cell resistance of the bulk, R_b_, interface layer, charge transfer reaction and diffusion process (Warburg impedance) with a single experiment. Two equivalent circuit models and Nyquist plots are depicted in Figure 6. SIE spectra can show the shape of a nearly perfect semicircle following the Debye response or incomplete depressed semicircle in the high-frequency region with non-Debye response, with an inclined spike in the low-frequency region due to the electrode polarization. In the first case, the R_b_ value can be determined by finding the intersection point of the semicircle and x-axis in the low-frequency region. The quantity of the R_b_ value of the electrical resistance in the second EIS spectrum can be estimated from the intersection point of the corrected centered semicircle on the Z_Re_ axis.

The matrix structure and conductive composition of the cement material have an influence on the high-frequency arc. The electrochemical reaction of the electrode and/or the behavior of the electrode–specimen interface can affect the low-frequency arc [23].

The bulk electrical resistance can be associated with the development of micro-cracks in the structure of the cementitious sample. The value of the bulk electrical resistance is significantly responsible for the state of health of the material. A decrease in bulk resistance indicates a rarefied microstructure with the higher amount of microcracks due to the higher expansion and vice versa [3]. It is known that a large expansion of the alkaline–silica reaction will cause more cracks [24].

**Figure 7:**
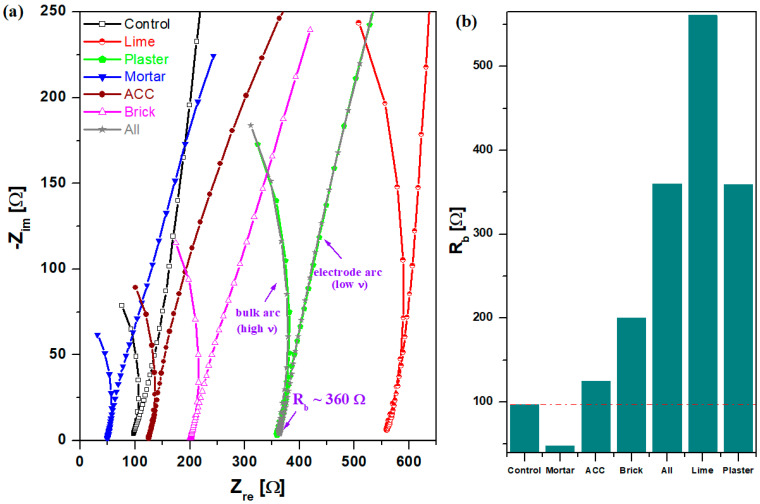
(**a**) Nyquist curves in an alkaline solution and (**b**) the bulk electric resistance, R_b_, as a function of composite–cement composition. The red line shows the value of the control specimen.

Figure 7 depicts Nyquist plots of the complex impedance obtained in NaOH solution of 1M concentration and further the compositional evolutions of bulk electric resistance, R_b_, for composite–cement and control cement materials. For the studied samples, the impedance spectra consist of two components: a semicircle in the high-frequency range due to the bulk arc and electrode arc (linear shape). The bulk arc at higher frequency is associated with the electrical properties of the composite–cement material. The electrode arc situated at lower frequency corresponds the polarization effect of the electrode/sample interaction. For the composite–cement materials, the values of the bulk resistance, R_b_, are improved considerably from 28.4% (ACC sample) at 476.4% (lime sample) exception, making the mortar–cement sample superior compared to the control. There is an increase in the R_b_ value of 270% for the All sample compared with the control cement, which can be linked to the smaller amounts of microcracks and to insignificant alkaline–silica reaction expansion.

Moreover, the semicircle diameter of the SIE spectrum shows also the overall resistance related to the ion transport in cement materials. The increase in impedance of the cement materials can be assigned to the denser pore structure, which inhibits the ion transport, and as result, the diameter of the semicircle of the Nyquist plot was increased [25]. In our study, for ACC, brick, plaster and All samples, the diameter of the high-frequency arc increases, then reaches the maximum value for the lime sample, and after that, decreases slightly for the mortar sample.

For the control cement and mortar sample, the bulk resistances were decreased from 97.3 at 48.2 Ω, attributed to cracks and the results of the alkaline–silicate reaction expansion. The mortar sample exhibits better conductivity than the cement material.

### 3.5. Cyclic and Sweep Linear Voltammetry

**Figure 8:**
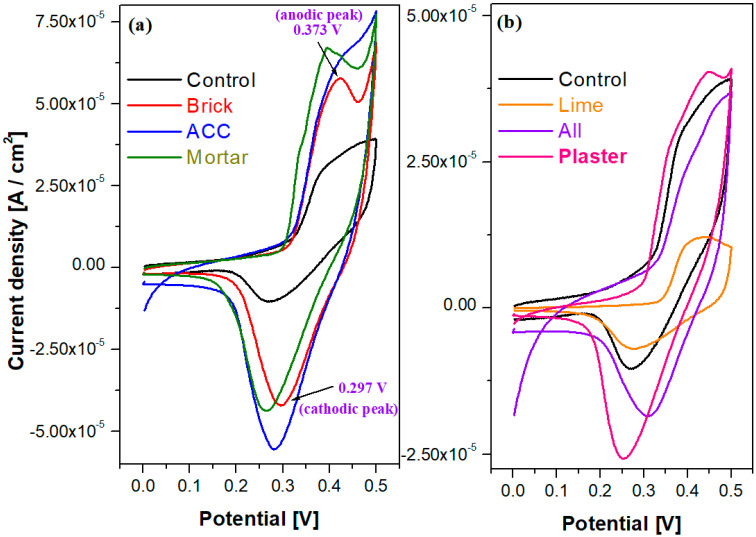

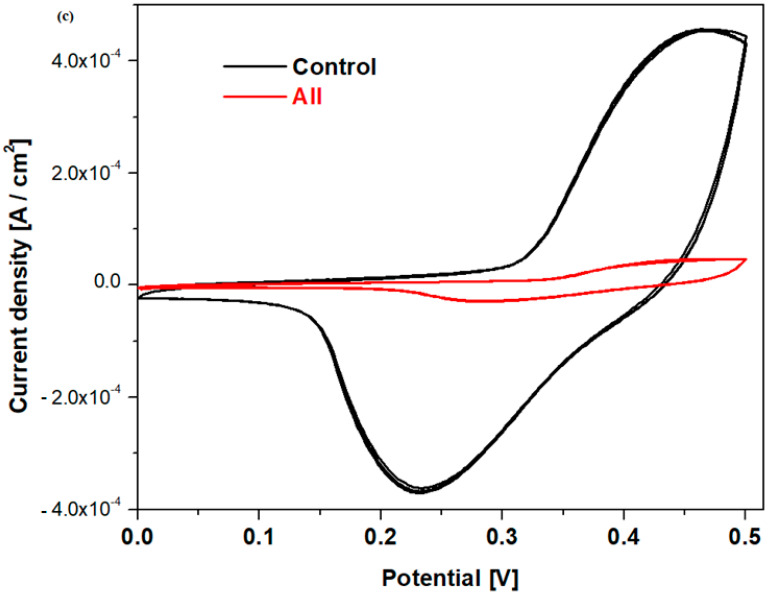
Cyclic voltammograms recorded in alkaline solution of composite–cement materials (control and all samples) using working electrode for one cycle (**a**,**b**) and after the scanning of the three cycles (**c**).

Further, the EIS results are correlated with cyclic voltammograms (CV) and sweep linear voltammetry (SLV) data. The cyclic voltammograms of the cementitious materials in the alkaline environment are shown in Figure 8. The shapes of cyclic voltammograms are consistent with a set of oxidation/reduction peaks. For the adding of composites such as mortar, ACC, brick and plaster in the cement material, the curves are very broad, anodic/cathodic peaks can be identified, and the current densities were increased compared with those of their analogues and the control sample. For example, the electrochemical behavior of the mortar–cement sample is characterized by the presence of an oxidation peak situated at 373 mV (corresponding to the oxidation process from the solution) and cathodic wave response at 297 mV (ΔE = 76 mV). The cyclic voltammogram scan after three cycles for the control and All samples showed irreversible processes (Figure 8c).

The current response was significantly increased and the waves during oxidation and reduction processes were improved for the samples Brick, ACC, Mortar, Plaster and All. By doping with lime in the cement material, there are significant differences between these two plots, and the current densities are also decreased compared with those obtained with their analogues.

**Figure 9:**
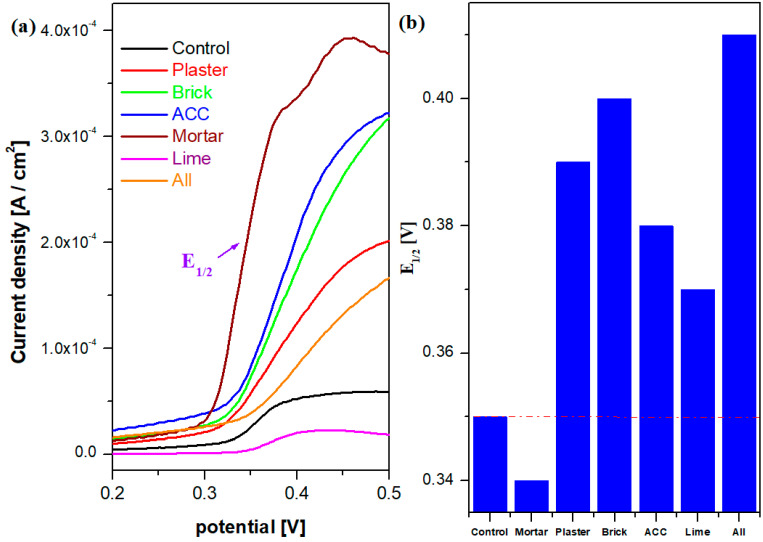
(**a**) Sweep linear voltammograms and (**b**) the values of the half-wave potentials, E_1/2_, versus composite–cement composition (the red line indicates the value of the control sample).

Figure 9 shows the SLV plots and the composition variation of cementitious materials as a function of 1/2 half-wave potentials, E_½_. As expected, in the composite with lime, the oxidation signal is considerably lower than that recorded with their analogues. Further, the value of the ½ half-wave potential attains the minimum value for the mortar composite, indicating the best electrochemical performance and better reversibility of the voltammogram.

### 3.6. Evolution of Bulk Electrical Resistance after 3, 7, 14 and 28 Days in NaOH Solution

In order to understand the effect of the composites on the composition of reaction products during the alkali–silica reaction, two cement samples, namely the control cement and composite–cement material, were prepared and immersed in a natrium hydroxide solution of 1M concentration at room temperature for 3, 7, 14 and 28 days.

In the present study, we explored a novel cell that uses natrum hydroxide electrolyte in a three-electrode configuration consisting of a working electrode, such as the composite–cement sample, and counter/reference electrodes to evaluate the effect of alkaline immersion.

The Nyquist data of the impedance can be also used to determine the bulk electrical resistance, R_b_, during the accelerated test of the alkali–silica reaction at 3, 7, 14 and 28 days in NaOH solution of 1M concentration. The intersections at the higher-frequency arc and the lower-frequency line correspond to the resistances of cement materials, R_b_ [25].

**Figure 10:**
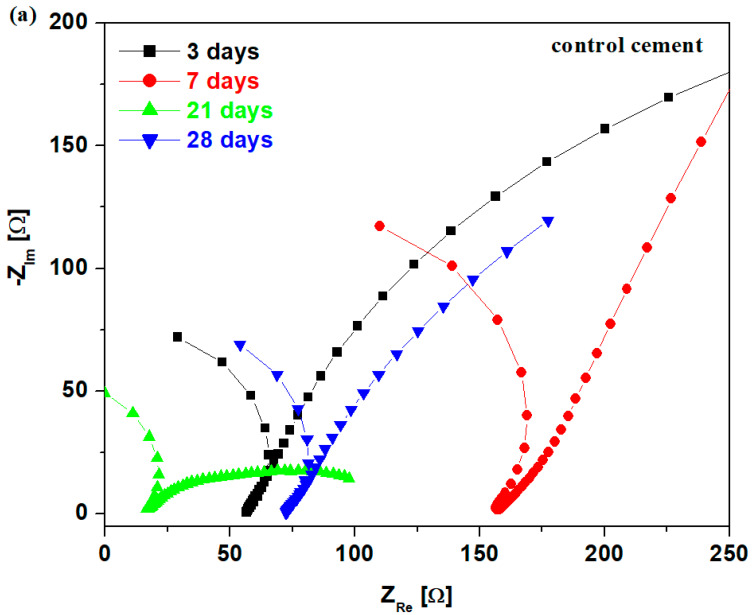

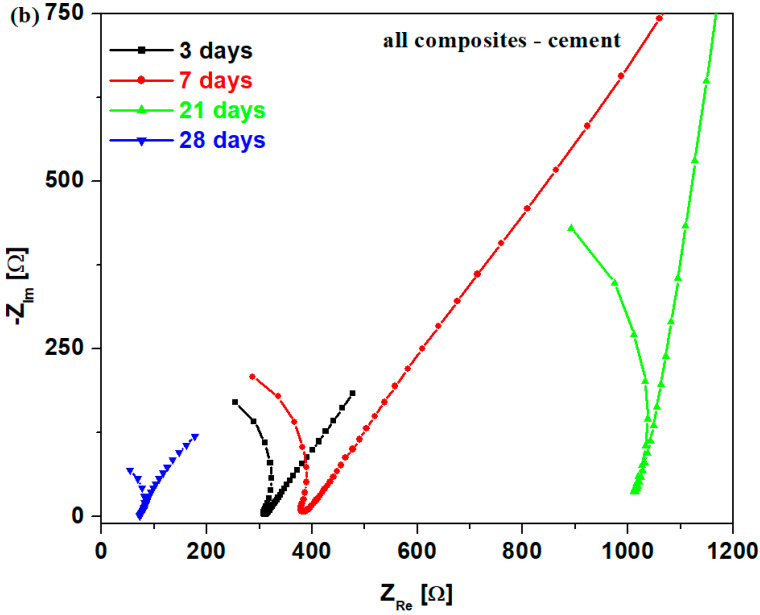

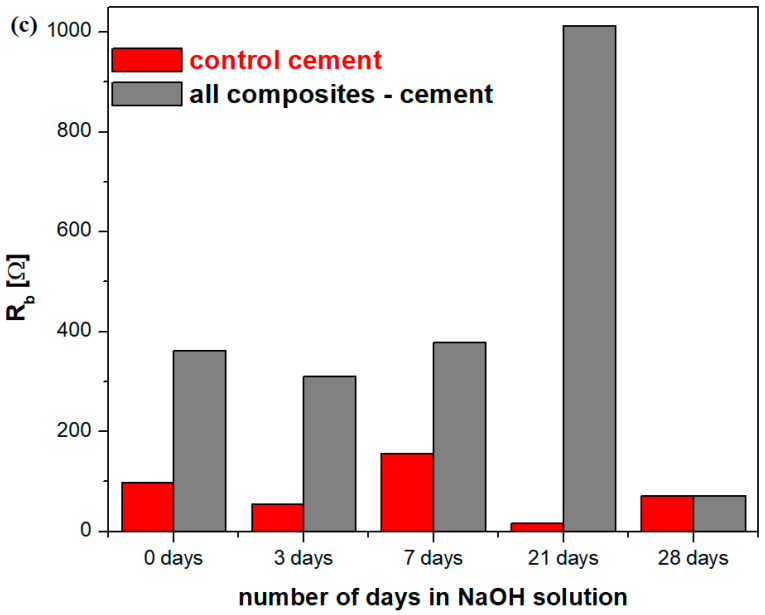
Nyquist plots of (**a**) control cement and (**b**) all composites—cement sample. (**c**) Resistance values recorded after some days of alkaline environment of the studied cementitious materials.

The EIS spectra and the change in bulk resistance, R_b_, at different days of immersion of the control cement and all composite–cement materials are depicted in Figure 10. The Nyquist diagrams are composed of real and imaginary impedances and also consist of bulk and electrode arc at varied frequencies. At the intersection in the x-axis of the two arcs, the bulk electrical resistance, R_b_, can be determined.

The impedance spectra in the complex plane indicate random displacements when comparing the control cement at 3 days with those at 7, 14 and 28 days of alkaline immersion. A trend was observed of shifting towards the right after 7 days, then towards the left after 21 days, and finally, towards the right after 28 days. The value of bulk resistance is improved after 7 days, and after that, its values were decreased after more days of immersion. The reduction in the R_b_ value is attributed to the formation of more microcracks because the expansion processes of the alkali–silicate reactions are largely intensified.

For the all composite–cement material, the impedance plots shift gradually to the right and the values of bulk resistances increase continually with the increase in days up to 21 days immersed in NaOH solution; after that, there is an opposite trend, as well as a drastic decrease in the R_b_ value at 28 days. This decrease in the bulk resistance at 28 days of immersion is linked to the expansion of the alkali–silica reaction and the formation of microcracks. The micro-cracks can create discontinuous paths, changing the continuous structure of the material, which results in the decrease in bulk resistance.

The structure of control cement remains unaffected by alkali–silicate reactions up to 7 days in alkaline immersion, while for the composite–cement material, it was unaffected for up to 21 days, respectively. For the control sample, all bulk resistance values are smaller than those of the composite–cement, suggesting the results of expansion of the alkali–silica reaction. All values of the bulk resistances at 0, 3, 7 and 21 days are more enhanced for cementitious material containing all composites compared to the control sample, showing a higher chemical stability of composite–cement material under alkaline attack.

The lime sample has the lowest value for particle size of the CaCO_3_ crystalline phase. It is known that calcium carbonate is responsible for the rate of hydration of cement and strength development in concrete [26]. The resistance value is very high for lime among the other composite–cement sample due to the presence of CaCO_3_ crystalline phase with smaller particle sizes, which improve the pore structure and mechanical resistance under disturbing external factors.

The Nyquist plots (shape and circuit) can be correlated with the response of other oxide materials or the electrical double layer of the electrode interface [27,28].

In brief, the cementitious material including different types of mixed composites behaves without the evolution of the alkali–silica reactions and without cracking for up to 21 days, while the control material lasts only 7 days. All values of electrical resistances of the control sample are lower, indicating the acceleration of the alkali–silica process and the formation of more micro- and macrocracks in the material. The abrupt increase in the electric resistance from 3 days to 7 days and from 7 days to 21 days, respectively, can be linked to the continuous hydration of the cementitious materials doped with composites.

### 3.7. Linear/Circular Economy of C&D Waste

The replacement of natural raw materials can minimize and reduce the effects on the environment and permit the cement industry to become a major player in C&D waste recycling. In conclusion, this study evidences the possibility of the circular economy of the spent materials used in construction.

The construction industry consumes up to 40% of global raw materials, generates over 40% of waste and emits over 25% of carbon dioxide into the atmosphere. In Europe, the construction industry provides jobs for more than 18 million people. An increase of 60% in building resource management is expected to generate an additional USD 1.6 trillion annually. Rapid urbanization and providing a working or living space have major contributions in this area. The economic benefits will continue only if the construction resources are consumed efficiently and sustainably [29].

Generally ingrained in the construction industry is the approach “take, make, dispose”, known as a linear economy, which creates the problem of an unsustainable economic situation. A linear economy is an approach to capitalization on building materials used for the purpose of construction, and at the end of life of the building, they are discarded. They are designed and assembled for a single use without exploring the advantages of recycling materials back into the system. Linear economics focuses on the limited lifetime of resources without considering the end of life of the products.

The cumulative problems of the linear economy have created many concerns among governments, professionals in construction and decision makers regarding the need to find a sustainable way to prevent environmental consequences, consumption of natural resources and generation of waste. Consequently, the circular economy has emerged as a veritable initiative to promote a sustainable built environment with an increased efficiency for construction resources and waste minimization. The goal of a circular economy is to minimize waste, and the materials from which a product is made, at the end of its life, will be reintegrated into the economy as much as possible.

The circular economy paradigm has awakened public interest around the world as an innovative and significant attempt to conserve finite resources, to reduce waste and to abandon the linear economy. Although the circular economy is still in the incipient stages of the management of construction and demolition waste, the scientific contributions of the circular economy agenda are growing in the construction industry.

**Figure 11:**
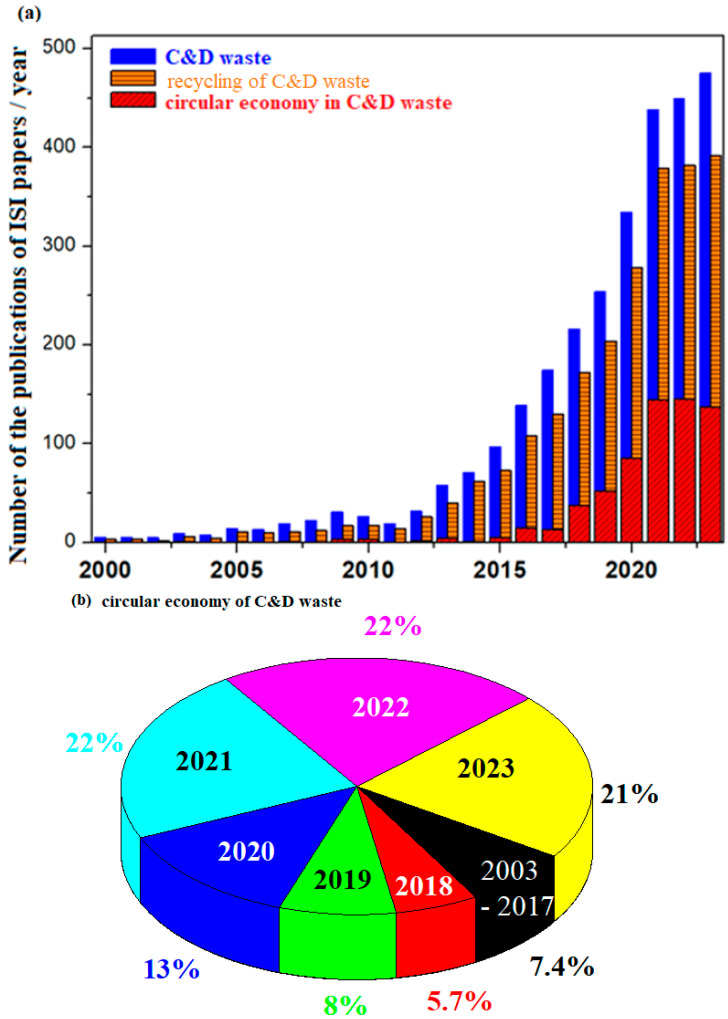
(**a**) The evolution of scientific publications on construction and demolition waste, their recycling and circular economy. (**b**) The main years with the highest percentage of publications on the circular economy of C&D waste.

The distribution of the number of publications per year analyzing the C&D waste, recycling and circular economy of C&D waste in the construction field is presented in Figure 11. For the publications until 2014, their number does not exceed 100 ISI-quoted articles per year in all domains. Then follow an almost linear growth until the year 2020, reaching 350 publications per year on the subject of C&D waste and 280 articles on C&D waste recycling, respectively. Between the years 2021 and 2023, the number of publications reaches the highest values, between 450 and 500 publications per year for the C&D waste topic and nearly 400 articles for C&D waste recycling, respectively.

Regarding the term of circular economy of C&D waste, the first publication appears in 2003, and the second in 2007, and until the year 2015, they are summarized in a maximum of five articles. An almost linear growth is reflected in the diagram until the year 2019, and in recent years, an increase of almost 140% is achieved compared to the year 2007. For example, in the year 2021, the number of publications increased, with 59 ISI articles compared to the year 2020, and down one publication compared to 2022. From the 648 publications between the years 2003 and 2023, distributed throughout scientific journals, the concept of the circular economy in the construction field is explained. There is a sudden upward trend in the study of this topic between the years 2018 and 2023 and a significant interest in the last three years, over 21%, increasing by almost four times compared with the annual publication trend of the year 2018 (Figure 11b).

Inspection of these developments indicates an increased interest over the years, and a major increase in published articles on the topic of C&D waste can be observed. The number of publications increases continually in the field, so that in the year 2023, the number of publications is high, with increases of almost 7.24 times (for C&D waste), 8.80 times (for C&D waste recycling) and 33.35 times (for circular economy of C&D waste), respectively, compared with 2013. In conclusion, interest related to these topics has grown significantly in recent years.

## 4. Conclusions

This paper provides a systematic review on the use of composites as an alternative for cement material. Six different types of composites were prepared by the nwet synthesis method using as raw materials construction and demolition waste and glass powder. The effects of varied composites on structure and mechanical/electrical properties of cement material and on the mitigation of the alkali–silica reaction were also detailed. X-ray diffractograms show that the structure of nanocomposite–cement materials consists of di- and tri-calcium silicate, calcium carbonate and smaller amounts of calcium hydroxide.

The NMR data show that the internal water is gradually consumed and the area of different relaxation peaks tends to gradually change. The T2 relaxation time shifts from longer relaxation time to shorter relaxation time at 28 days after preparation. This modification corresponds with the evolution of the cement hydration process. A trend of shifting of the peak corresponding to bounded water indicates the mobility of the components of composite–cement material.

The incorporation of composites in the cement material improves the Vickers hardness after aging for one year after their preparation.

The bulk electrical resistance of composite–cement samples increases compared to the control, while the value of R_b_ of the mortar sample was decreased, suggesting the presence of microcracks.

In the cyclic voltammograms, the intensities of the oxidation and reduction peaks increase for the mortar and brick samples.

When the composite–cement samples are exposed to the accelerated test of the alkali–silica reaction, an improvement in the bulk electrical resistances was noticed from 3 days to 21 days, which could be due to the smaller expansion, the continuous hydration process and the pozzolanic reaction of the cement material. For the control sample, the reduction in bulk resistance after 7 days could be due to the larger expansion, the formation of more micro-cracks and the deterioration of the microstructure. Based on our results, we can conclude that studied nanocomposites play an important role in the deleterious effect of expansion of the cement material.

This approach can be applied in the future for other types of cementitious materials which use highly reactive composite as a replacement for higher cement content in concrete or mortar, respectively.

The success of preparing and using new composites based on construction and demolition waste opens new directions towards the circular economy of spent building materials for the construction field, in particular, the cement industry.

## Figures and Tables

**Table 1:** Description of prepared samples.

Notation of Prepared Composites	Raw Materials Powders Used in Synthesis of Composites	Notation of Prepared Composite–Cement	Composition of the Composite–Cement	
L	Mixture of glassy and lime waste	Lime	2.5 weight % of cement is substituted by composites containing C&D waste	L
P	Mixture of glassy and plaster waste	Plaster		P
M	Mixture of glassy and mortar waste	Mortar		M
A	Mixture of glassy and autoclaved aerated concrete waste	ACC		A
B	Mixture of glassy and brick waste	Brick		B
T	Mixture of glassy, lime, plaster, autoclaved aerated concrete, brick, mortar	All		T
		Control	100% cement	

## Data Availability

Data will be made available on request.
